# Editor’s Page: What Ever Happened to the Physical Examination?

**DOI:** 10.1016/j.shj.2023.100265

**Published:** 2024-01-17

**Authors:** Anthony DeMaria

**Affiliations:** Judy and Jack White Chair in Cardiology, Sulpizio Cardiovascular Center, University of California, San Diego, La Jolla, California, USA

The history and physical examination have been the foundation of clinical medicine since the beginning of time. Those considered among the most brilliant and talented physicians have traditionally been those who could reach a diagnosis prior to any laboratory testing, even if it were contained in a differential. For years physicians have prided themselves on the ability to hear a murmur, feel a liver/spleen, or detect a hyperactive reflex. When I took my Cardiology Board examination it consisted of both a patient examination exam and a written test.

As the years have progressed there has been continuous development of powerful technological tools that have provided more information than was available from the physical examination. Laboratory analysis, nuclear medicine, and particularly imaging have enabled diagnoses to be established more accurately and quantitatively and with more certainty than the physical exam. Technology has often provided evidence of presence and severity of disease that could not be obtained by the physical. Not surprisingly, this has led to a greater reliance on tests than the physical exam in patient management.

The prominence of laboratory testing has invariably led to a diminution in the skill of performing the physical examination. Numerous studies have documented reduced skills in cardiac auscultation among physicians at all levels, but more prominent among those who were trained more recently.^1^^,^^2^ One can assume that the ability in palpation and percussion etc., are even more attenuated. This has led to a concern that the pendulum may have overly swung with the physical exam largely being abandoned, even in regard to those aspects that are very clinically useful.

This Editor’s page was prompted by a recent experience in which the role of the physical exam was largely neglected. The case was that of an elderly gentleman admitted to a general medicine service for multiple systemic complaints and low blood pressure. A systolic murmur was heard and an echo was ordered. The echo revealed a low transaortic valve gradient with a calculated valve area of moderate stenosis but with extremely calcified and immobile leaflets. A cath was performed that revealed an aortic valve area in the critical range. Great debate followed as to whether the aortic valve was majorly responsible for the patient’s condition and if he was too high a risk for even a percutaneous intervention. Ultimately a transcatheter aortic valve replacement was successfully performed.

The discussion at cath conference initially expressed surprise that the echo valve area was in error. It was also debated as to whether the aortic stenosis was in fact a major contributor to the patient’s condition. It was not until the end of the discussion that someone suggested that findings on physical such as a diminished carotid artery upstroke, murmur radiation, and evidence of left ventricular hypertrophy might have been of value in corroborating the role of the aortic stenosis.

This case reminded me of my own experience during a long career in cardiology. Echo was just being introduced during my fellowship, and prior to that the assessment of AS was by the physical exam and ultimately cardiac catheterization. The clinical skills were such that the severity of AS by cath was usually predicted by physical exam. The advent of echo provided a noninvasive method to quantify AS that was usually, but not always, accurate compared to cath. The question then was whether any patient required cath before surgery. So highly was the physical valued that the consensus was that a cath should be performed only in those patients in whom there was a difference between the assessment by physical exam and echo. As the years have passed, the reliance upon echo, and other imaging, has predominated, not that echo has become more accurate, but that the physical exam skills have atrophied. This has resulted in the aforementioned case in which the physical exam was barely described, not applied to assessing the possible clinical significance, and surprise was expressed at the variance between the echo and cath findings.

No one would deny that the advances made in laboratory testing and especially imaging have made an enormous contribution to the clinical care we can deliver. I, for one, would hate to go back to having to rely only on the physical exam to assess structural or other forms of heart disease. Even physicians of my vintage recognize the ability of imaging to provide information that is not available from the physical exam. Nevertheless, I am worried that the pendulum has indeed overswung and that even the useful information that the physical exam can provide is being neglected. Physical findings can identify an abnormality or confirm its presence and severity as determined by other methods. At a time when concern is expressed about the possible overutilization of noninvasive imaging, a good physical exam can go a long way to determine the necessity for such procedures.

Although I do not know how, I want to lobby for a greater emphasis upon acquiring and applying physical examination skills by cardiovascular specialists. Perhaps we should require that a full, detailed exam be described at all case presentations and in all imaging requests, and not just “a systolic murmur” or even worse “a heart murmur.” I am not so naive as to think we will ever return to the likes of Wenkebach who is said to have recognized his periodicity from observation of the jugular vein impulse. But I believe I am justified in mourning what has become of the physical examination and urging a resuscitation.

## Funding

The author has no funding to report.

## Disclosure Statement

The author reports no conflict of interest.
